# Development and preliminary validation of the Brief Self-Compassion Inventory

**DOI:** 10.1371/journal.pone.0285658

**Published:** 2023-05-12

**Authors:** Kelly Chinh, Wei Wu, Shelley A. Johns, Patrick V. Stutz, John H. McGrew, Catherine E. Mosher

**Affiliations:** 1 Department of Psychology, Indiana University-Purdue University Indianapolis, Indianapolis, Indiana, United States of America; 2 Seattle Division, VA Puget Sound Healthcare System, Seattle, Washington, United States of America; 3 Department of Medicine, Indiana University School of Medicine, Indianapolis, Indiana, United States of America; 4 Regenstrief Institute, Inc., Center for Health Services Research, Indianapolis, Indiana, United States of America; University of Concepcion Faculty of Medicine: Universidad de Concepcion Facultad de Medicina, CHILE

## Abstract

Research and clinical interest in self-compassion has grown due to its associations with physical and mental health benefits. Widely used measures of self-compassion have conceptual and psychometric limitations that warrant attention. The purpose of this project was to develop a new self-compassion measure, the Brief Self-Compassion Inventory (BSCI), and test its psychometric properties. We developed items for the BSCI based on theory, prior research, and expert and cancer patient feedback. The BSCI was then tested with adults diagnosed with breast, gastrointestinal, lung, or prostate cancer (*N* = 404). Confirmatory factor analysis suggested a unidimensional structure, and internal consistency reliability was excellent. Construct validity of the BSCI was established through its correlations with psychological variables hypothesized to be related to self-compassion, such as mindfulness, acceptance of cancer, and other coping strategies. Furthermore, measurement invariance testing of the BSCI indicated that it could be used across patients of varying genders, cancer types, and stages of illness. In conclusion, the 5-item BSCI was determined to be psychometrically sound and suitable for use with adults of varying genders, cancer types, and stages of disease. The measure warrants testing with other medical and nonclinical populations.

## Introduction

In recent years, self-compassion and its associations with physical and psychological health have garnered attention [1]. Most studies of self-compassion have been conducted using the Self-Compassion Scale (SCS) or its short-form (SCS-SF) [1,2]. These scales include three facets, each with a positive and a negative dimension [2–4]. The first facet is *self-kindness*, defined as being gentle and compassionate towards oneself, versus *self-judgment*, which involves reacting harshly to inner experiences. The second facet is *common humanity*, a shared understanding of human suffering, versus *isolation*, the feeling that one is alone in one’s imperfection or suffering. The third facet, *mindfulness*, refers to being aware of the present-moment experience of suffering while keeping such experiences in perspective. Thus, one avoids extremes of dissociation or *overidentification*, which involves getting caught up in difficult thoughts and feelings. SCS items measuring both compassionate and uncompassionate behavior have often been combined to form three facets and one overall self-compassion construct [1,2,4]. However, researchers have noted inflated correlations between this overall construct and psychopathology due to negatively worded items and have critiqued the theory underlying the measure [5,6].

Self-compassion is theoretically linked to psychological processes commonly targeted in behavioral interventions. One process is mindfulness, defined as a facet of self-compassion [2–4] or as a separate but interrelated construct [7,8]. A process negatively associated with self-compassion is psychological inflexibility, which refers to behavior driven by internal reactions (e.g., unwanted thoughts and feelings) rather than personal values [9]. Such behavior patterns are thought to be at the core of most human suffering [10]. Another process negatively associated with self-compassion is cognitive fusion, or the tendency for behavior to be overly controlled by thoughts [11]. Self-compassion is also conceptually related to values-based living, or behaving in a manner that matches stated values [10].

Other psychological processes related to self-compassion include rumination and avoidant and active coping. Rumination refers to the tendency to dwell on aspects of situations that are upsetting, which hinders active problem solving [12,13]. Avoidant coping includes mental and behavioral disengagement from the stressor and denying its reality [14]. Self-compassion has been linked to less avoidant and more active coping in chronic illness populations [15].

Cancer patients are one chronic illness population for which self-compassion is central to coping and adjustment [16]. Among cancer populations, self-compassion has been linked to lower distress and better quality of life [16–18]. In addition, growing evidence supports the feasibility and efficacy of compassion-focused and mindfulness-based interventions for cancer patients [19–22]. However, research is needed to examine self-compassion as a mediator of improved psychological adjustment to cancer.

The measurement of self-compassion, with most studies using the SCS or SCS-SF, has been critiqued [6]. Researchers have noted that individuals without college-level literacy may have difficulty reading the SCS [3]. Many possible factor structures for the SCS have been proposed and tested in diverse samples with differing conclusions about the fit of these structures with the data [23–27]. Some analyses of the SCS’s structure show two separate factors grouped by positive and negative items [3,24,28,29]. Given that most studies use a total SCS score, researchers have proposed separate scales for positive and negative items (self-compassion and self-criticism) [3,24,28,29]. This approach may help address artificially inflated associations of the SCS with psychopathology [6]. Indeed, only the negative SCS items accounted for a significant portion of the variance in associations with psychopathology [5].

Several alternative self-compassion measures have been developed, though evidence for their reliability and validity is limited [30]. These measures include the Relational Compassion Scale [31], the Sussex-Oxford Compassion Scales [32], and the Compassionate Engagement and Action Scales [33]. The Relational Compassion Scale includes one 3-item subscale assessing self-kindness; mindfulness and common humanity are not assessed [31]. The Sussex-Oxford Compassion Scales include a 20-item measure of self-compassion, conceptualized as five facets (e.g., recognizing suffering, tolerating uncomfortable feelings) that do not include mindfulness [32]. The Compassionate Engagement and Action Scales include two self-compassion subscales assessing engagement with distressed feelings and taking action to cope with distress [33]. While aspects of mindfulness are included in scale items, self-kindness and common humanity are not assessed.

To address the limitations of existing self-compassion measures, we developed a new measure of self-compassion, the Brief Self-Compassion Inventory (BSCI). Following a review of theories of compassion and self-compassion [2–4,30,34–36] and existing self-compassion measures [2,30,37], we decided to refrain from using negatively worded items and assess the same three positive facets of self-compassion as the SCS while updating the definition of mindfulness. Whereas the SCS mindfulness subscale refers to balanced awareness of present-moment experiences of suffering, the BSCI assesses mindful acceptance, defined as approaching ongoing thoughts and feelings with openness. These internal experiences are viewed as an ever-changing and natural part of life. This conceptualization reflects typical definitions of mindfulness in the psychological literature [38–40]. We maintained Neff’s [4] definitions of self-kindness and common humanity in our new measure and conceptualized the three facets of self-compassion as highly correlated protective factors with respect to physical and mental health.

Our measure was tested in patients diagnosed with solid tumors. We first conducted an exploratory factor analysis (EFA) and a parallel analysis to assess the factor structure of our original 15-item Self-Compassion Inventory (SCI). We then conducted confirmatory factor analyses (CFAs) to test two different factor structures for the 15-item SCI, including a single-factor model with an overall self-compassion factor (Model 1) and a hierarchical model with three factors indicating a higher-order overall self-compassion factor (Model 2). Model 1 was based on results of our EFA and parallel analysis, and Model 2 was grounded in aspects of Neff’s conceptual model [4]. We then selected items to form the 5-item BSCI and conducted a CFA to determine whether it had the same factor structure as the SCI. We also evaluated the BSCI’s internal consistency and construct validity. We expected moderate associations between the BSCI and both positive psychological variables (i.e., mindfulness, quality of life, peaceful acceptance of cancer, active coping, and progress in values-based living) and negative psychological variables (i.e., depressive symptoms, anxiety, rumination, denial, struggle with illness, psychological inflexibility, cognitive fusion, and obstruction in values-based living) based on theory [8,10–12]. Additionally, we examined whether the BSCI had smaller correlations with negative psychological variables (e.g., anxiety, depressive symptoms, rumination) compared to the SCS-SF total score and smaller correlations with anxiety and depressive symptoms compared to negative items of the SCS-SF. Finally, we examined the measurement invariance of the BSCI across cancer stages, cancer types, and genders.

## Materials and methods

### Generation of initial item pool

Initially, 12 items were generated based on a conceptual model of self-compassion (i.e., self-kindness, mindful acceptance, and common humanity) [4] and existing self-compassion measures. A five-point response scale was selected with choices ranging from 1 = “not at all” to 5 = “very much.” The items were reviewed by three doctoral-level experts in self-compassion and mindfulness. Based on expert feedback and cognitive interviews with 10 cancer patients (see S1–S3 Appendices for cognitive interview methods and results), three items were added and other items were altered prior to psychometric testing. Expert and patient feedback primarily focused on wording changes to improve clarity. The preliminary measure for psychometric testing included 15 items.

### Participants and procedures

All study procedures were approved by the Indiana University’s institutional review board (IRB). Participants were adults with a diagnosis of breast, gastrointestinal, lung, or prostate cancer recruited from a public hospital, an academic cancer center, and affiliated clinics in Indianapolis, Indiana from February to October 2018. Eligible patients were identified through institutional cancer registries. They were either ≥3 weeks post-diagnosis of stage IV cancer or had completed active treatment ≥6 months ago for stage I or II cancer. Participants showed no evidence of severe cognitive impairment based on a cognitive screener [41]. To ensure representation of demographic subgroups based on the National Cancer Institute’s Surveillance, Epidemiology, and End Results (SEER) data [42], purposive sampling was conducted by race, ethnicity, and gender. Eligible patients received mailed recruitment packets followed by a phone call for eligibility screening and verbal informed consent, which the research assistant documented in writing. The IRB approved a verbal informed consent process for this minimal risk study because the research assistant was not approaching patients in person. The survey was administered either online through REDCap or a mailed paper copy per each participant’s preference. Participants received a $25 gift card for completing the survey.

### Measures

Except for the SCI and BSCI, all study measures have been previously tested with cancer populations with evidence of reliability and validity.

### Demographic and medical information

Cancer information, age, and gender were retrieved from patients’ medical records. Patients self-reported other demographics and whether they had been diagnosed with or treated for 12 medical comorbidities in the last three years [43].

#### Self-compassion (SCI/BSCI)

The initial measure or SCI had five items for each potential subscale—self-kindness, common humanity, and mindful acceptance. Participants rated each item with reference to the past two weeks on a 5-point Likert scale ranging from 1 = “not at all” to 5 = “very much.” This scale was selected to parallel Patient-Reported Outcomes Measurement Information System (PROMIS) measures [44]. All items were positively worded; thus, higher scores represented greater self-compassion. In follow-up analyses, five items were selected to form the BSCI.

#### Self-compassion (SCS-SF)

The 12-item Self-Compassion Scale-Short Form (SCS-SF) [37] was also administered. The SCS-SF contains six subscales—self-kindness, self-judgment, common humanity, isolation, mindfulness, and over-identification—and a 5-point Likert scale with opposing anchors of “almost never” and “almost always.” The six negatively worded items in the SCS-SF measure were reverse-scored, and all items were summed to compute a global self-compassion score.

#### Anxiety and depressive symptoms

The 4-item PROMIS Anxiety and Depression measures were used to assess anxiety and depressive symptoms, respectively [45,46]. Both measures use a 5-point Likert scale with responses ranging from “never” to “always.”

#### Quality of life

The McGill single item scale [47] was used to assess quality of life. Patients were asked to rate their overall quality of life, considering all aspects of their life, on an 11-point scale (0 = “very bad” to 10 = “excellent”).

#### Mindfulness

Subscales of the Five Facet Mindfulness Questionnaire-Short Form (FFMQ-SF) [48] were used to assess mindfulness. The FFMQ-SF consists of five 3-item subscales: Observing, Describing, Acting with awareness, Nonjudging of inner experience, and Nonreactivity to inner experience. Items are rated on a 5-point Likert scale (1 = “never or very rarely true” to 5 = “very often or always true”). The latter three subscales were administered in our survey because they were most predictive of mental health and symptom outcomes in prior research with cancer survivors [49].

#### Psychological inflexibility

Psychological inflexibility was assessed with the 7-item Acceptance and Action Questionnaire-II (AAQ-II) [50]. Participants were asked to rate the degree to which painful thoughts and feelings interfered with daily life and coping on a 7-point Likert scale ranging from “never true” to “always true.”

#### Values-based living

The 10-item Valuing Questionnaire (VQ) [51] was used to assess obstruction and progress in values-based living. Half of the questionnaire’s items are negatively worded (Obstruction subscale), and the other half are positively worded (Progress subscale). Respondents rated how true each item was for them on a 7-point Likert scale ranging from “not true at all” to “completely true.”

#### Cognitive fusion

The 7-item Cognitive Fusion Questionnaire (CFQ) [11] was used to assess cognitive fusion, or the tendency to become entangled in thoughts that lead to overregulation of one’s behavior. The CFQ contains statements about participants’ thoughts. Participants rated how true each statement was for them on a 7-point Likert scale ranging from “never true” to “always true.”

#### Acceptance of cancer

The 12-item Peace, Equanimity, and Acceptance in the Cancer Experience (PEACE) [52] measure was used to assess patients’ acceptance of their cancer. The measure contains two subscales—Struggle with Illness (7 items) and Peaceful Acceptance (5 items). All items are rated on a 4-point Likert scale ranging from “not at all” to “to a large extent.”

#### Denial and active coping

Two of the 14 subscales from the Brief COPE [14]—Denial and Active Coping—were used to assess coping responses to cancer-related stress. Each subscale contains two items and uses a 4-point Likert scale (1 = “I haven’t been doing this at all” to 4 = “I’ve been doing this a lot”).

#### Rumination

The Rumination subscale of the Rumination-Reflection Questionnaire (RRQ) [13] was used to assess rumination. Participants rated their level of agreement with each item on a 5-point Likert scale ranging from “strongly disagree” to “strongly agree.”

### Data analyses

#### Preliminary analyses

Descriptive statistics were calculated to characterize patients’ demographic and medical information and other characteristics. The data were also examined for normality, linearity, skewness, and kurtosis. Data were considered skewed and kurtotic using guidelines of >|3| and >|10|, respectively [53].

#### Psychometric testing of the SCI/BSCI

First, we examined whether items were performing poorly based on the following criteria: 1) low factor loadings (i.e., <0.40); 2) low item-total correlations (i.e., <0.30) [54]; 3) one category received less than 5% of responses; or 4) more than 80% endorsed the highest or lowest category (i.e., there is a ceiling or floor effect) [55]. We considered eliminating items based on these numerical criteria while maintaining coverage of the main facets of self-compassion—self-kindness, common humanity, and mindful acceptance.

EFA [56] and parallel analysis [57,58] were performed to examine the factor structure of the 15-item SCI in Mplus Version 8 [59] (see S4 Appendix for description of methods and results). Results suggested that there was one factor according to model fit information and interpretability of factor structures. We then conducted CFAs to test two models for the 15-item SCI, including a single-factor model with an overall self-compassion factor and a hierarchical model with three factors indicating a higher-order overall self-compassion factor. A description of the CFAs and tests of internal consistency reliability and validity for the SCI are found in the S4 Appendix.

Given evidence for the unidimensionality of the SCI, the five items with 1) the highest item-total correlations/loadings and 2) providing sufficient content coverage for the target construct were selected to form the BSCI. A single-factor CFA was performed to examine whether the BSCI was also unidimensional. The robust full information maximum likelihood estimation method (RFIML) was used for the CFA to account for the nonnormality of ordinal items and missing data [60]. RFIML has been shown to be an appropriate approach to dealing with ordinal missing data [60]. Eight percent of participants had missing observations on some of the items. Model fit was determined using several indices, including the chi-square test statistic, comparative fit index (CFI), root-mean-square error of approximation (RMSEA), and standardized root mean square residual (SRMR). Although model fit guidelines vary, acceptable model fit was defined as: (1) a non-significant *χ*^2^ statistic; (2) CFI>0.90; (3) RMSEA<0.08; and (4) SRMR<0.06 [61]. Another popular approach to analyzing ordinal data is the weighted least squares mean and variance adjusted (WLSMV) method. We did not use WLSMV in this study because it could produce less accurate chi-square test statistics than RFIML given the presence of missing data [62].

Internal consistency reliability of the BSCI was examined by computing Cronbach’s alpha and the omega coefficient using SPSS version 27 (IBM Corp., Armonk, NY, USA). To assess the BSCI’s construct validity, theory-driven correlations between BSCI scale scores (sum of items) and main study variables were examined. The analyses were conducted in Mplus using RFIML. We also computed correlations between main study variables and SCS-SF total scores, SCS-SF positively worded items (summed), and SCS-SF negatively worded items (summed). We then examined whether the BSCI had smaller correlations with negative psychological variables, such as anxiety and depressive symptoms and rumination, compared to the SCS-SF total score. Additionally, we examined whether the BSCI had smaller correlations with anxiety and depressive symptoms compared to negatively worded SCS-SF items. To determine whether the correlations were significantly different, we first converted the correlations into *z-*scores using Fisher’s *r*-to-*z* transformation and then used a *z*-test to test the significance [63].

#### Measurement invariance

Given our focus on creating a generalizable measure, we tested measurement invariance of the BSCI across cancer stages, cancer types, and genders. Invariance models were tested in stepwise order, starting from the least restrictive level to the most restrictive level. The first level, the configural invariance model, imposes the same factor structure simultaneously across the groups without any parameter constraints. The second level is the metric invariance (weak invariance) model, where factor loadings are constrained to be equal across the groups. The third level is scalar invariance (strong invariance) where both factor loadings and intercepts are constrained to be equal across the groups. Finally, the fourth and most restrictive level is the strict invariance model where factor loadings, intercepts, and error variances are all constrained to be equal across the groups. Again, RFIML was used for all the models and scaled Satorra-Bentler chi-square difference tests [64] were conducted to compare the models. A nonsignificant chi-square difference test indicated that the more restricted model was preferred.

## Results

Of the 701 patients who received recruitment materials, 109 were not reached, 29 were ineligible, 99 declined, and 60 did not complete the survey or were omitted from analyses (e.g., survey returned with significant missingness), leading to a sample size of 404. Sample characteristics are found in Table 1. Results for the initial 15-item SCI, including item selection, factor structure, reliability, and validity, can be found in the S4–S10 Appendices. Code for all analyses in this paper and the appendices can be found in the S11 Appendix.

**Table 1:** Sample characteristics (*N* = 404).

Characteristic	*N* (%)	*M* (*SD*)	Range
Age	--	62.6 (11.1)	28.0–89.0
Female	205 (50.7)		
Race^a^			
White	325 (80.4)		
Black/African American	60 (14.9)		
Native American or Alaska Native	8 (2.0)		
Asian American	10 (2.5)		
Other	8 (2.0)		
Ethnicity			
Non-Hispanic/Latinx	374 (92.6)		
Hispanic/Latinx	14 (3.5)		
Missing	16 (4.0)		
Relationship status			
Single	48 (11.9)		
Married/living with partner	275 (68.1)		
Divorced/separated	53 (13.1)		
Widowed	27 (6.7)		
Missing	1 (0.0)		
Education level			
Elementary / Some high school	24 (5.9)		
High school graduate	88 (21.8)		
Some college or technical school	122 (30.2)		
College graduate	94 (23.3)		
Graduate school	74 (18.3)		
Missing	2 (0.5)		
Employment status			
Employed full-time	124 (30.7)		
Employed part-time	34 (8.4)		
Retired	160 (39.6)		
Unemployed, looking for work	3 (0.7)		
Unemployed due to disability	67 (16.6)		
Other	14 (3.5)		
Missing	2 (0.5)		
Annual household income			
< $21,000	67 (16.6)		
$21,000 - $39,999	75 (18.6)		
$40,000 - $65,999	69 (17.1)		
$66,000 - $105,999	92 (22.8)		
≥ $106,000	91 (22.5)		
Missing	10 (2.5)		
Cancer type			
Breast	101 (25)		
Gastrointestinal	101 (25)		
Lung	102 (25.2)		
Prostate	100 (24.8)		
Cancer stage			
Early (Stage I or II)	203 (50.2)		
Advanced (Stage IV)	201 (49.8)		
Time since diagnosis (years)	--	3.3 (3.0)	0.1–23
Cancer treatments			
Surgery	324 (80.2)		
Chemotherapy	194 (48.0)		
Radiation therapy	147 (36.4)		
Chemoradiation	31 (7.7)		
Hormone therapy	135 (33.4)		

^a^Participants were able to select multiple responses for this question. Thus, counts do not total to 404 or 100%.

### Item selection and factor structure

The five items with the highest item-total correlations were retained for the BSCI. The five selected items also met face validity, reflecting the main facets of self-compassion—self-kindness, common humanity, and mindful acceptance. Given that a single factor structure was best for the 15-item measure, we fit a single factor model to the BSCI. The model had a better model fit (SRMR = 0.03, CFI = 0.98, RMSEA = 0.07) than all models tested with the SCI. The BSCI and SCI scale scores were highly correlated (*r* = 0.94). Item-total correlations and inter-item correlations were also examined (see Table 2), and no problematic items were found. All standardized loadings exceeded the recommended cut-off of 0.40 (range = 0.76–0.83, see Table 2). Internal consistency reliability was excellent (α = 0.90; ω = 0.90).

**Table 2:** Descriptive statistics, inter-item correlations, and standardized loadings for the Brief Self-Compassion Inventory items.

Item	1	2	3	4	5	Mean	SD	Item-Total Correlation	Standardized Loadings
1. When I had difficult feelings, I realized that these feelings would change over time.	--					3.77	1.08	0.71	0.76
2. When I faced a challenge, I reminded myself that challenges are a part of every human life.	0.64	--				3.98	1.05	0.77	0.83
3. I recognized that my struggles are also experienced by others.	0.65	0.70	--			4.01	1.05	0.76	0.82
4. I was able to soothe myself during times of stress.	0.57	0.63	0.60	--		3.67	1.00	0.74	0.80
5. I accepted my painful thoughts and feelings as a natural part of life.	0.57	0.63	0.63	0.73	--	3.85	1.04	0.75	0.81

All correlations are significant at *p* < .01.

### Construct validity

To test the construct validity of the BSCI, correlations were computed between the BSCI scale score and all main study variables (see Table 3). The BSCI was positively associated with mindfulness (i.e., acting with awareness [*r* = 0.21, *p* < .001], nonjudging [*r* = 0.16, *p* = .003], and nonreactivity [*r* = 0.21, *p* < .001]), quality of life (*r* = 0.40, *p* < .001), peaceful acceptance of cancer (*r* = 0.39, *p* < .001), active coping (*r* = 0.16, *p* = .002), and progress in values-based living (*r* = 0.43, *p* < .001). In addition, significant negative associations were found between the BSCI and depressive symptoms (*r* = -0.37, *p* < .001), anxiety (*r* = -0.36, *p* < .001), rumination (*r* = -0.32, *p* < .001), denial (*r* = -0.15, *p* = .001), struggle with illness (*r* = -0.30, *p* < .001), psychological inflexibility (*r* = -0.39, *p* < .001), cognitive fusion (*r* = -0.36, *p* < .001), and obstruction in values-based living (*r* = -0.36, *p* < .001). A moderate positive correlation was also found between the BSCI and the existing SCS-SF (*r* = 0.55, *p* < .001).

**Table 3:** Correlations for assessing the construct validity of the Brief Self-Compassion Inventory.

	*n*	BSCI	SCS-SF Total	SCS-SF Positive Items	SCS-SF Negative Items	*z* ^a^	*p*	*z* ^b^	*p*
Acting with awareness	396	0.21*	0.44*	0.20*	0.51*	--	--	--	--
Nonjudging	391	0.16*	0.45*	0.18*	0.54*	--	--	--	--
Nonreactivity	397	0.28*	0.15*	0.35*	-0.05	--	--	--	--
Quality of life	403	0.40*	0.47*	0.27*	0.48*	--	--	--	--
Peaceful acceptance of illness	397	0.39*	0.45*	0.34*	0.40*	--	--	--	--
Active coping	401	0.16*	0.09	0.26*	-0.08	--	--	--	--
Values-based living—Progress	398	0.43*	0.57*	0.56*	0.40*	--	--	--	--
Depressive symptoms	397	-0.37*	-0.50*	-0.30*	-0.52*	2.25	0.02	2.64	0.01
Anxiety symptoms	397	-0.36*	-0.49*	-0.26*	-0.52*	2.23	0.03	2.80	0.01
Rumination	390	-0.32*	-0.68*	-0.38*	-0.72*	6.92	0.00	--	--
Denial	397	-0.15*	-0.31*	-0.13*	-0.35*	2.38	0.02	--	--
Struggle with illness	398	-0.30*	-0.52*	-0.26*	-0.58*	3.75	0.00	--	--
Psychological inflexibility	396	-0.39*	-0.61*	-0.30*	-0.67*	4.17	0.00	--	--
Cognitive fusion	397	-0.36*	-0.70*	-0.38*	-0.75*	6.88	0.00	--	--
Values-based living—Obstruction	402	-0.36*	-0.61*	-0.32*	-0.65*	4.49	0.00	--	--
SCS-SF Total	390	0.55*	--	--	--	--	--	--	--

BSCI, Brief Self-Compassion Inventory; SCS-SF, Self-Compassion Scale–Short Form.

*p < .01.

^a^Statistically comparing correlations between negative variables and the BSCI vs. the SCS-SF.

^b^Statistically comparing correlations between depressive and anxiety symptoms and the BSCI vs. negative items of the SCS-SF.

Further evidence for the construct validity of the BSCI was obtained. Compared to associations with the SCS-SF total score, associations were significantly smaller between the BSCI and depressive symptoms (*z* = 2.26, *p* = 0.02), anxiety (*z* = 2.64, *p* = 0.03), rumination (*z* = 6.92, *p* < .001), denial (*z* = 2.38, *p* = 0.02), struggle with illness (z = 3.74, *p* < .001), psychological inflexibility (*z* = 4.17, *p* < .001), cognitive fusion (*z* = 6.88, *p* < .001), and obstruction in values-based living (*z* = 4.49, *p* < .001) (see Table 3). Additionally, compared to associations with negatively worded SCS-SF items, associations were smaller between the BSCI and depressive symptoms (*z* = 2.64, *p* = .008) and anxiety (*z* = 2.80, *p* = .005).

### Invariance testing of the BSCI

Measurement invariance testing results are found in Table 4.

**Table 4:** Measurement invariance across cancer stage, cancer type, and gender.

	*χ*^2^ (*df*)	CFI	RMSEA [90% CI]	Sequential Model Comparison *χ*^2^ (*df*)	*p*
Cancer Stage^a^					
Configural	22.24 (10)	0.97	0.08 [0.03–0.12]	--	
Metric	29.15 (14)	0.97	0.07 [0.04–0.11]	3.89 (4)	0.42
Scalar	38.05 (18)	0.96	0.07 [0.04–0.11]	9.45 (4)	0.051
Strict	43.81 (25)	0.96	0.06 [0.03–0.09]	5.88 (7)	0.55
Cancer Type^b^					
Configural	43.95 (20)	0.96	0.11 [0.07–0.15]	--	
Metric	63.18 (32)	0.94	0.10 [0.06–0.13]	14.56 (12)	0.27
Scalar	82.46 (44)	0.93	0.09 [0.06–0.12]	16.94 (12)	0.15
Strict	93.05 (65)	0.95	0.07 [0.03–0.09]	16.50 (21)	0.74
Gender					
Configural	27.87 (10)	0.97	0.09 [0.05–0.14]	--	
Metric	35.21 (14)	0.96	0.09 [0.05–0.12]	3.00 (4)	0.56
Scalar	41.70 (18)	0.95	0.08 [0.05–0.11]	3.84 (4)	0.43
Strict	52.94 (25)	0.95	0.07 [0.05–0.10]	11.51 (7)	0.12

CFI, comparative fit index; RMSEA, root mean square error of approximation.

^a^Early (stage I and II) and advanced (stage IV) cancer.

^b^Breast, gastrointestinal, lung, and prostate cancer types.

#### Cancer stages

For the early and advanced-stage cancer groups, the configural model demonstrated a good fit (CFI = 0.97, RMSEA = 0.08). The chi-square difference test between the configural and metric models was nonsignificant (Δ*χ*^2^ = 3.89, *p* = 0.42). There was no statistically significant difference between the metric and scalar models either (Δ*χ*^2^ = 9.45, *p* = 0.051) or between the scalar and strict models (Δ*χ*^2^ = 5.97, *p* = 0.99), indicating that strict invariance was satisfied.

#### Cancer types

For the breast, gastrointestinal, lung, and prostate cancer groups, the configural model demonstrated an adequate fit (CFI = 0.96, RMSEA = 0.11). The chi-square difference tests were nonsignificant between the configural and metric models (Δ*χ*^2^ = 14.56, *p* = 0.27), metric and scalar models (Δ*χ*^2^ = 16.94, *p* = 0.15), and scalar and strict models (Δ*χ*^2^ = 16.54, *p* = 0.74), indicating that strict invariance had been met.

#### Gender

For male and female participants, the configural model demonstrated adequate fit (CFI = 0.97, RMSEA = 0.09). Once again, the difference tests showed nonsignificant differences between the configural and metric models (Δ*χ*^2^ = 3.00, *p* = 0.56), metric and scalar models (Δ*χ*^2^ = 3.84, *p* = 0.43), and scalar and strict models (Δ*χ*^2^ = 11.49, *p* = 0.12), indicating that strict invariance held.

## Discussion

This study aimed to develop and evaluate the psychometric properties of a new self-compassion measure, the BSCI. Findings support the BSCI’s unidimensional factor structure and excellent psychometric properties. Furthermore, results suggest strict invariance of the BSCI across cancer stages, cancer types, and genders, indicating psychometric equivalence for use across subgroups. To date, most self-compassion research has used the SCS or SCS-SF, which have conceptual and psychometric limitations [5,6,65]. Other self-compassion measures have limited evidence of validity [30]. Overall, the BSCI addresses limitations of existing self-compassion measures and will help advance self-compassion research.

The unidimensional factor structure of the BSCI may have emerged for multiple reasons. In contrast to the SCS [2], our items reflected a standard conceptualization of mindfulness that may be more highly correlated with other facets of self-compassion. The omission of negatively worded items also may have contributed to our measure’s unidimensional structure. Although negatively worded items are thought to prevent acquiescence bias (the tendency to over-endorse items), these types of items have not been found to prevent this bias [66]. In fact, negatively worded items may lead to errors due to careless responding or elicit a different response pattern than positively worded items [67,68]. Research suggests that if 10% show careless responses to such items, a unidimensional structure is likely to be rejected [68].

Construct validity of the BSCI was supported through its correlations with theory-driven psychological variables. Consistent with theory [39], the BSCI was positively associated with the mindfulness facets of nonreactivity, nonjudging, and acting with awareness. Further evidence of construct validity included the BSCI’s positive associations with peaceful acceptance of cancer and progress in values-based living and negative associations with their counterparts, struggle with illness and obstruction in values-based living. Findings are consistent with theory suggesting that acceptance of cancer requires one to turn towards this difficult experience with kindness rather than attempting to avoid it [69]. Theory also suggests that greater self-compassion will result in prioritizing actions that benefit the self, such as living more consistently with personal values [10,70]. In addition, greater self-compassion on the BSCI was related to higher levels of active coping, less denial, and better quality of life, which converges with prior research [71].

Regarding “negative” psychological variables, the BSCI was inversely associated with rumination, psychological inflexibility, and cognitive fusion. The inverse link between self-compassion and rumination is consistent with prior research with cancer populations [17]. In addition, people with low self-compassion may judge or criticize their thoughts and feelings rather than recognize the universality of such experiences. According to contextual behavioral theory, this judgment of internal experiences is associated with greater cognitive fusion or entanglement with thoughts and psychological inflexibility [10]. This inflexibility emerges when people attempt to avoid difficult experiences, resulting in behaviors inconsistent with personal values (e.g., avoiding family gatherings in fear of others asking about their cancer).

Consistent with predictions, associations with “negative” psychological constructs (e.g., rumination, denial) were 1.6 to 2.2 times as large for the SCS-SF total score compared to the BSCI, with most associations being close to twice as large. This pattern of findings appeared to result from negatively worded items in the SCS-SF. Indeed, negatively worded items in the SCS-SF had significantly larger correlations with anxiety and depressive symptoms compared to the BSCI. Thus, the BSCI addresses an important limitation of the SCS-SF and SCS, as items do not have inflated correlations with psychopathology.

Strict measurement invariance was established for the BSCI across cancer stages, cancer types, and genders, suggesting that observed differences in self-compassion between subgroups most likely reflect true group differences rather than the result of biased measurement. Thus, the BSCI is acceptable for use with adults with varying cancer stages (early, advanced), cancer types (breast, gastrointestinal, lung, and prostate cancer), and genders and can be used to make meaningful comparisons between these subgroups.

Limitations of this study should be noted. Although purposive sampling based on National Cancer Institute data ensured more representation of racial and ethnic minority individuals, the majority were non-Hispanic white, reflecting the demographics of our recruitment sites in Indiana. In addition, this study was cross-sectional, which did not allow for an examination of test-retest reliability. Furthermore, to reduce participant burden, other constructs for the validity analyses were not assessed, such as self-criticism and compassion for others. Finally, levels of anxiety and depressive symptoms in our sample were low compared to cancer patient norms [72]. Among cancer patients, lower distress is correlated with lower symptom burden and better adjustment [73].

## Conclusions

In conclusion, improving the measurement of self-compassion is necessary for advancing science. By following gold-standard practices for measure development and testing [44,74,75], this study produced a new 5-item self-compassion measure, the BSCI, with robust psychometric properties. The BSCI was found to be acceptable for use across samples of varying cancer stages, cancer types, and genders. Next steps include testing the BSCI with more culturally diverse cancer populations as well as other medical and nonclinical populations. In addition, the BSCI could be tested as a mediator of compassion-focused or mindfulness-based interventions’ effects on psychological and physical health outcomes. Overall, use of this brief measure will reduce participant burden, improve the validity of self-compassion measurement, and allow for examination of theory underlying compassion-focused interventions.

## Supporting information

S1 Appendix Cognitive interviewing methodology. (DOCX)

S2 Appendix Semi-structured cognitive interview guide. (DOCX)

S3 Appendix Major themes from cognitive interviews and resulting changes to self-compassion measure. (DOCX)

S4 Appendix Statistical methods and results for psychometric testing of the 15-item Self-Compassion Inventory. (DOCX)

S5 Appendix Descriptive statistics for Self-Compassion Inventory items (*N* = 372). (DOCX)

S6 Appendix Inter-item pearson and polychoric correlations for the Self-Compassion Inventory (*N* = 404). (DOCX)

S7 Appendix Exploratory Factor Analysis (EFA) and parallel analysis results for the 15-item Self-Compassion Inventory. (DOCX)

S8 Appendix Goodness of fit indices for proposed factor structures for the 15-item Self-Compassion Inventory. (DOCX)

S9 Appendix Item loadings for the Self-Compassion Inventory. (DOCX)

S10 Appendix Correlations for assessing the construct validity of the 15-item Self-Compassion Inventory. (DOCX)

S11 Appendix Code for data analyses. (DOCX)
