# Supplementary material for: Development and preliminary validation of the Brief Self-Compassion Inventory
Source: PLoS One. 2023 May 12;18(5):e0285658. doi: 10.1371/journal.pone.0285658 (PMC10180635; doi:10.1371/journal.pone.0285658)
Supplement: S1 Appendix — (DOCX) [file pone.0285658.s001.docx]

**S1 Appendix. Cognitive interviewing methodology**

**Participants**

Qualitative feedback was obtained from 10 cancer patients on the 12 preliminary items of our self-compassion measure. The number of interviews was consistent with recommendations for cognitive interviews provided by NIH Patient-Reported Outcomes Measurement Information System (PROMIS) researchers [1]. Patients were recruited from hematologic and gastrointestinal cancer clinics at an academic cancer center in Indianapolis, Indiana. To ensure gender and age diversity, purposive sampling was used to enroll approximately equal numbers of men and women as well as individuals <65 years and ≥65 years of age.

Patient eligibility was determined through medical chart review and consultation with the attending oncologists. Eligible patients were: (1) diagnosed with hematological or gastrointestinal cancer; (2) 18 years of age or older; (3) fluent in English; (4) not exhibiting significant psychiatric or cognitive impairment as indicated by a score ≥4 on a 6-item cognitive screener [2]; and (5) not enrolled in hospice care. Five participants were advanced cancer patients receiving anti-cancer therapy or supportive care, and five were cancer survivors who were at least six months post-treatment.

**Procedure**

Study procedures were approved by the Indiana University’s institutional review board. Following oncologist approval, a research assistant approached the patient before or after their oncology appointment. The research assistant met with interested patients in a private clinic room to complete the written informed consent process.

After obtaining consent, participants were asked to complete a paper survey consisting of demographic items and the 4-item PROMIS Anxiety and Depression measures [3]. Then the research assistant conducted an audiotaped cognitive interview to obtain participants’ feedback on the new self-compassion measure [4]. The interview guide is found in the S2 Appendix. The research assistant held up a card with each item from the new self-compassion measure printed in large letters, as well as a separate card with the measure’s response options / anchors printed in large letters. The research assistant then asked the participant to read it aloud and provide their thoughts and first impressions of each question before answering. In accordance with cognitive interviewing principles, participants were asked follow-up questions about the language, comprehensibility, ambiguity, and relevance of each item using a semi-structured interview guide [5]. Such questions included, “What were you thinking of when you answered that question?,” “How did you go about deciding on which answer to pick?,” and “How easy or hard was it to tell the difference between each choice on the scale?” The cognitive interviews lasted about 30 minutes on average. Following completion of the interview, the participant was given a $25 gift card.

**Measures**

**Demographic and medical information**

The pre-interview survey assessed the following demographic information: race, ethnicity, marital status, education level, income, and employment status. The following variables were extracted from medical records: age, gender, cancer type and stage, date of diagnosis, and cancer treatments received.

**Anxiety and depressive symptoms**

Anxiety and depressive symptoms were assessed using the 4-item PROMIS Anxiety and Depression measures [3, 6]. Internal consistency reliabilities were excellent (anxiety α=0.93, depression α=0.95) [3]. Both measures have shown evidence of reliability and validity with cancer patients [7-10].

**Data analyses**

Descriptive statistics (e.g., means, standard deviations, frequencies) were calculated to characterize patients’ demographic and medical information and anxiety and depressive symptoms.

Qualitative interview data were analyzed using a basic content analysis, which is a systematic coding and categorization process to make inferences from the data [11]. Our qualitative analysis included the following phases: (1) interview transcription by trained research assistants; (2) reading and coding of interview transcripts and categorization of concepts; and (3) generation of descriptive themes [1]. Themes were used to identify potential alterations to the measure.

Using an inductive approach, coders first read transcripts of the audiotaped interviews before creating codes to categorize participants’ feedback on the items [12]. The team of four coders included two clinical health psychologists with experience in qualitative data analysis, a clinical psychology graduate student, and a research assistant. The transcripts were divided, and each was reviewed by at least two coders who independently developed codes and met regularly to discuss them and reach a consensus. Next, themes were generated during team meetings by categorizing recurring codes. Inter-relationships among themes were also examined. The salience of thematic findings was assessed by evaluating the extent to which both coders had drawn similar conclusions during their independent review and synthesis of the coded interview data and the degree to which themes recurred across interviews [12]. The themes were then checked to ensure that they were internally consistent and distinguishable from one another. Alterations were made to our self-compassion measure based on study findings (see S3 Appendix).

The sample was predominantly non-Hispanic white (8/10) with a mean age of 64 (SD=11). The sample was balanced in terms of gender, early vs. advanced stage diagnoses, and type of cancer. Average time since the cancer diagnosis was 5.4 years (SD=3.9 years). On average, participants reported low levels of anxiety (mean=5.4, SD=1.4) and depressive symptoms (mean=5.1, SD=2.0).

**References**

1. DeWalt DA, Rothrock N, Yount S, Stone AA. Evaluation of item candidates: the PROMIS qualitative item review. Med Care. 2007;45(5 Suppl 1):S12-21.

2. Callahan CM, Unverzagt FW, Hui SL, Perkins AJ, Hendrie HC. Six-item screener to identify cognitive impairment among potential subjects for clinical research. Med Care. 2002;40(9):771-81.

3. Pilkonis PA, Choi SW, Reise SP, Stover AM, Riley WT, Cella D. Item banks for measuring emotional distress from the Patient-Reported Outcomes Measurement Information System (PROMIS®): depression, anxiety, and anger. Assessment. 2011;18(3):263-83.

4. Cella D, Riley W, Stone A, Rothrock N, Reeve B, Yount S, et al. The Patient-Reported Outcomes Measurement Information System (PROMIS) developed and tested its first wave of adult self-reported health outcome item banks: 2005-2008. J Clin Epidemiol. 2010;63(11):1179-94.

5. Willson S, Miller K. Data collection. In: Miller K, Willson S, Chepp V, Padilla J-S, editors. Cognitive interviewing methodology. Hoboken, NJ: Wiley; 2014. pp. 15-33.

6. Choi SW, Reise SP, Pilkonis PA, Hays RD, Cella D. Efficiency of static and computer adaptive short forms compared to full-length measures of depressive symptoms. Qual Life Res. 2010;19(1):125-36.

7. Jensen RE, Potosky AL, Moinpour CM, Lobo T, Cella D, Hahn EA, et al. United States population-based estimates of Patient-Reported Outcomes Measurement Information System symptom and functional status reference values for individuals with cancer. J Clin Oncol. 2017;35(17):1913-20.

8. Adams RN, Mosher CE, Rand KL, Hirsh AT, Monahan PO, Abonour R, et al. The Cancer Loneliness Scale and Cancer-related Negative Social Expectations Scale: Development and validation. Qual Life Res. 2017;26(7):1901-13.

9. Cella D, Choi S, Garcia S, Cook KF, Rosenbloom S, Lai J-S, et al. Setting standards for severity of common symptoms in oncology using the PROMIS item banks and expert judgment. Qual Life Res. 2014;23(10):2651-61.

10. Wagner LI, Schink J, Bass M, Patel S, Diaz MV, Rothrock N, et al. Bringing PROMIS to practice: Brief and precise symptom screening in ambulatory cancer care. Cancer. 2015;121(6):927-34.

11. Hsieh H-F, Shannon SE. Three approaches to qualitative content analysis. Qual Health Res. 2005;15(9):1277-88.

12. Lune H, Berg BL. An introduction to content analysis. In Qualitative research methods for the social sciences. 9th ed. Harlow, England: Pearson Education; 2017. pp. 181-200.
