# Supplementary material for: Development and preliminary validation of the Brief Self-Compassion Inventory
Source: PLoS One. 2023 May 12;18(5):e0285658. doi: 10.1371/journal.pone.0285658 (PMC10180635; doi:10.1371/journal.pone.0285658)
Supplement: S2 Appendix — (DOCX) [file pone.0285658.s002.docx]

**S2 Appendix. Semi-structured cognitive interview guide**

Introduction and Procedures

Once we get started, do I have your permission to audio record our conversation like we discussed? Your answers will be kept anonymous and will only be used for this study.

I’d like to talk with you about a short questionnaire that we are developing. We’re going to discuss some questions that will ask about your thoughts, feelings, and actions over the past 2 weeks. In particular, the questions will ask you to think back to times when you weren’t feeling your best to see how you handled those tougher times*.* (*Pause and gauge for level of understanding*. *At the end of each of the following paragraphs, participants will have the opportunity to interject, ask questions, etc.*)

The questions aren’t perfect, and we would like your help in improving them. Each question is printed here on a notecard. I’ll hand you one card at a time, and I’d like you to read the question aloud. Then, immediately start thinking out loud by saying what’s going through your head as you decide on your answer. (*Pause and gauge for level of understanding and address/restate parts of above procedure as needed.*)

After you choose your answer on the scale, I’ll ask you a few more questions before we move on to the next card. Does that make sense? (*Pause and* *gauge for level of understanding* *and address/restate parts of above procedure as needed.*)

During our conversation, please feel free to point out any words or questions that are unclear, difficult to read, don’t make sense to you, or seem to be missing something. If a question or word has ANY problems with it, voice your thoughts as soon as you have them. There are no right or wrong answers in this interview—we want to know exactly what YOU think and how YOU understand the question. So if you ask me a question during the interview, I may not be able to answer.

Do you have any questions before we start? (*Pause and* *gauge for level of understanding* *and address/restate parts of above procedure as needed.*)

(*Begin audio recording here.*)

Here’s the first question.

(*Hand participant the first notecard to be read aloud, and encourage thinking aloud until they arrive at their answer.)*

Follow-up Verbal Probes

1. (***SKIP if patient gave clear line of thinking during think-aloud****.*) What were you thinking of when you answered that question? What was going through your head?
2. (***SKIP if patient cited specific instance or example****.)* If possible, please describe a specific time or experience that you thought back to when you answered that. (*Probe for more specific details and rationale as needed.*)
3. How might your answer change if it asked about the past **30 days** instead of the past **2 weeks**? What about the past **7 days**? How would that timeframe make it easier or harder to choose an answer or think of a relevant experience?
4. For this question, how easy or hard was it to tell the difference between each choice on the scale? (*e.g. “Quite a bit” versus “Very much” & “A little bit” versus “Somewhat”*) *For discrepancies between answer choice and rationale*: “Okay, so you chose the answer ___, but then told me that _______.”
5. **Which words** in the question might be seen as unclear or confusing, either to you or to others? How could it be reworded? How else could the question be improved to make more sense?

Retrospective Probes (*to be asked after all items discussed*)

1. Finally, what else could we do to improve these questions when we use them in the future with other people?
2. (***SKIP if patient’s answers clearly reflected interpretation***) How did you interpret these questions overall as a survey? Did they come across as cancer-specific, or did they seem to be more generally referring to life?

Other Potential Probes

- Can you tell me in your own words what the question means to you?
- Was the question easy or hard to answer for the “past **2 weeks**”? Why?
- When you read the words “past **2 weeks**” which specific days did you think of (e.g., from which day to which day)?
- How did you choose between some of the answer choices (e.g., “Quite a bit” versus “Very much” & “A little bit” versus “Somewhat”)?
- You chose (*quote their answer*) as your answer. What does (*quote their answer*) mean to you?
- If you could change the answers or scale, what would you change?
- Did you think mostly about particular experiences on specific days, or more generally over the last **2 weeks**? (*if specific days/events*): Can you tell me more about what made you think back to those specific times?

**Preliminary Self-Compassion Measure for Cognitive Interviews**

Please respond to each item by marking one box per row.

| **In the past 2 weeks…** | | **Not at all** | **A little bit** | **Somewhat** | **Quite a bit** | **Very much** |
| --- | --- | --- | --- | --- | --- | --- |
| 1 | I was kind to myself even when I was going through a hard time. | 1 | 2 | 3 | 4 | 5 |
|  | | | | | | |
| 2 | Knowing that others have faced challenges similar to mine gave me comfort. | 1 | 2 | 3 | 4 | 5 |
|  | | | | | | |
| 3 | I noticed my difficult feelings without being overwhelmed by them. | 1 | 2 | 3 | 4 | 5 |
|  | | | | | | |
| 4 | When I noticed my weaknesses, I remembered that nobody is perfect. | 1 | 2 | 3 | 4 | 5 |
|  | | | | | | |
| 5 | I forgave myself for my mistakes. | 1 | 2 | 3 | 4 | 5 |
|  | | | | | | |
| 6 | I accepted my thoughts and feelings without needing to change them. | 1 | 2 | 3 | 4 | 5 |
|  | | | | | | |
| 7 | Even though I’ve failed before, I was able to let go of my past. | 1 | 2 | 3 | 4 | 5 |
|  | | | | | | |
| 8 | I recognized that I was not alone in my struggles. | 1 | 2 | 3 | 4 | 5 |
|  | | | | | | |
| 9 | I allowed myself to experience my painful thoughts and feelings instead of trying to avoid them. | 1 | 2 | 3 | 4 | 5 |
|  | | | | | | |
| 10 | When I had difficult feelings, I realized that these emotions would change. | 1 | 2 | 3 | 4 | 5 |
|  | | | | | | |
| 11 | I reminded myself that others experience times of difficulty like I do. | 1 | 2 | 3 | 4 | 5 |
|  | | | | | | |
| 12 | I was patient and understanding towards myself when facing challenges. | 1 | 2 | 3 | 4 | 5 |
