# Supplementary material for: Development and preliminary validation of the Brief Self-Compassion Inventory
Source: PLoS One. 2023 May 12;18(5):e0285658. doi: 10.1371/journal.pone.0285658 (PMC10180635; doi:10.1371/journal.pone.0285658)
Supplement: S3 Appendix — (DOCX) [file pone.0285658.s003.docx]

**S3 Appendix. Major themes from cognitive interviews and resulting changes to self-compassion measure**

| **Theme 1**: Participants took issue with the phrase “gave me comfort” in the item, “Knowing that others have faced challenges similar to mine gave me comfort.” |
| --- |
| “I do not like the thought of others having to face challenges with their health or life.” |
| “It would be pretty bad on my part if I was finding comfort in the fact that others were facing challenges.” |
| **Result**: Item changed to “Knowing that others have faced challenges similar to mine gave me courage.” |
| **Theme 2**: Participants described different interpretations of the word “weaknesses” in the item, “When I noticed my weaknesses, I remembered that nobody is perfect.” |
| “It’d be hard to… specify everything that could be a weakness. It might have to be explained like... an ability to do things or not.” |
| “I think… that nobody is perfect. I’m looking at my weakness as part of my…well, having the cold. But with my cancer and stuff, my immune system is down. So I’m run down to begin with.” |
| **Result**: Item changed to “When I noticed my flaws, I remembered that nobody is perfect.” |
| **Theme 3:** In response to the item, “I realized that I was not alone in my struggles,” some participants based their answer on their social support system rather common humanity, which was our intended meaning of the item. |
| “I’m thinking that my family is going through this with me, and my friends… so I’m not out there without a support system.” |
| “There are other people that have had the experiences I have had, or other people that are rooting for me. There are other people that are praying for me, and I know that I’m not alone.” |
| **Result:** Item changed to “I recognized that my struggles are also experienced by others.” |
| **Theme 4:** Participants had strong reactions to the item, “I allowed myself to experience my painful thoughts and feelings instead of trying to avoid them.” |
| “… I’m not touchy-feely, so my feelings don’t come into it much… I don’t really have them, so it’s not a matter of avoiding them. It’s a matter of not having them. I don’t really have intrusive thoughts… I don’t really allow myself to experience. I just experience it. So it’s like... you’re asking if I gave myself permission.” |
| “I’ve really tried to put them out of my consciousness so that they don’t overwhelm me, they don’t rule me and I can go on… I [thought] about what happens if it [the cancer treatment] doesn’t work and where we’re going with that . . . And I, I, I just had to avoid that because… [choking up] I was at a point that I could not deal with it.” |
| **Result**: The word “allowed” was omitted, changing the item to “I experienced my painful thoughts and feelings instead of trying to avoid them.” |
| **Theme 5:** Participants had different interpretations of the item, “I noticed my difficult feelings without being overwhelmed by them.” |
| “‘Overwhelmed?’ See, I take the word ‘overwhelmed’ to mean that it is all I’m thinking about, and I’m being crippled by difficult feelings.” |
| “My interpretation of what you’re asking is that I’ve been emotional about my circumstance, and therefore my emotions could overwhelm me, or I could deal with it… I noticed my difficult feelings 'quite a bit.' But I was overwhelmed 'not at all.'” |
| **Result**: Item was changed to “I noticed my difficult feelings without dwelling on them.” |
| **Theme 6:** In response to the item, “When I had difficult feelings, I realized that these emotions would change,” some participants seemed to tie emotions to unchangeable situations. |
| “I don’t think things are going to change... [including] how I feel and my situation with cancer… I’m stage four... this is my fourth time dealing with it... I think cancer is gonna be something I am going to have to face and deal with every day for the rest of my life... My feelings of my illness and how it affects my life... my family, my work... the emotions I have because of it I don’t think are going to change.” |
| “Well, I do always feel bad about the fact that I hardly do anything around the house to help my wife out. I always feel bad about that. Guilty, you know. The thing is, it doesn’t change because… it's something that can't change. Well, I suppose if I somehow were to get rid of my back pain, then things would change, you know. I’d be able to do more.” |
| **Result**: To enhance item clarity, “over time” was added to the end, making the final item “When I had difficult feelings, I realized that these feelings would change over time.” |
