# Supplementary material for: Development and preliminary validation of the Brief Self-Compassion Inventory
Source: PLoS One. 2023 May 12;18(5):e0285658. doi: 10.1371/journal.pone.0285658 (PMC10180635; doi:10.1371/journal.pone.0285658)
Supplement: S4 Appendix — (DOCX) [file pone.0285658.s004.docx]

**S4 Appendix: Statistical methods and results for psychometric testing of the 15-item Self-Compassion Inventory**

**Statistical Methods**

Exploratory factor analysis (EFA) [1] and parallel analysis [2] were conducted to examine the factor structure of the 15-item SCI in Mplus Version 8 [3]. The robust full information maximum likelihood estimation method (RFIML) was used to account for the nonnormality of ordinal items and missing data [4]. For the EFA, we evaluated three solutions: one, two, or three factors based on model fit and interpretability of factor structures (e.g., factor loading patterns and correlations among factors). Model fit was determined using several indices, including the chi-square test, comparative fit index (CFI), root-mean-square error of approximation (RMSEA), and standardized root mean square residual (SRMR). Although model fit guidelines vary, acceptable model fit was defined as: (1) a non-significant *χ*^2^ statistic; (2) CFI>0.9; (3) RMSEA<0.08; and (4) SRMR<0.06 [5]. The Akaike information criterion (AIC) and Bayesian information criterion (BIC) were used to compare models, with lower values indicating a better fit. The parallel analysis was performed on both a Pearson correlation matrix using Mplus and a polychoric correlation matrix of the ordinal items using the random.polychor.pa package in R [6].

Confirmatory factor analyses (CFAs) were then performed in Mplus using RFIML to test two factor structures for the 15-item Self-Compassion Inventory (SCI). The EFA and parallel analysis supported a one-factor model, whereas Neff’s conceptual model of self-compassion [7] suggests a second-order model with three first-order factors loading onto one higher-order factor. Thus, we used CFA to test both models: (1) a single-factor model indicating an overall self-compassion factor and (2) a hierarchical model with the three factors indicating an overall self-compassion factor. The same indices of model fit and model fit guidelines were used, as noted above. AIC and BIC were used to compare models.

Internal consistency reliability of the SCI was examined using a Cronbach’s alpha coefficient in SPSS version 27 (IBM Corp., Armonk, NY). To assess the SCI’s construct validity, theory-driven correlations between SCI scale scores (sum of items) and main study variables were examined. The analyses were conducted in Mplus using RFIML. We also examined whether the SCI had smaller correlations with negative psychological variables, such as anxiety and depressive symptoms and rumination, compared to the Self-Compassion Scale-Short Form (SCS-SF). Additionally, we examined whether the SCI had smaller correlations with anxiety and depressive symptoms compared to negative items of the SCS-SF. To examine whether the correlations were significantly different, we first converted the correlations into *z-*scores using Fisher’s *r*-to-*z* transformation and then used a *z*-test to test the significance [8].

**Results**

**Item selection**

Good performance for SCI items was demonstrated through high item-total correlations for all items (see S5 Appendix). Additionally, each of the five response options was endorsed across all 15 items. Thus, no items were eliminated. Responses tended to be on the higher end of the response scale, with >50% of respondents selecting a 4 (quite a bit) or 5 (very much) for each item. Inter-item Pearson and polychoric correlations are found in the S6 Appendix.

**Factor structure**

Results of the EFA and the parallel analysis are shown in the S7 Appendix. In the EFA, we compared models with one, two, or three factors (with goemin rotation: an oblique rotation method). Although the models with more factors fit the data better (i.e., the 2-factor model was better than the 1-factor model, and the 3-factor model was better than the 2-factor model), there was high redundancy between the factors. For example, the two factors in the 2-factor model had a significantly high correlation of .77, and two factors from the 3-factor model had a significant correlation of .87. There were also items with cross-loadings, making the factors less distinguishable. The loadings for the third factor in the 3-factor model were all low (< .45), indicating that the third factor was not well defined. Based on these considerations, we selected the one-factor model. More importantly, parallel analysis on either a Pearson or Polychoric correlation matrix suggested one common factor underlying the items.

We then used CFA to test two models: (1) 1 first-order factor (unidimensional) and (2) 1 second-order factor, 3 first-order factors (see S8 Appendix). Models 1 and 2 had adequate fit to the data (Model 1: SRMR=0.05, CFI = 0.89, RMSEA=0.08; Model 2: SRMR=0.05, CFI=0.91, RMSEA=0.08). When comparing Models 1 and 2, the AIC and BIC statistics suggested that Model 2 (AIC=14370.94, BIC=14563.01) may be superior to Model 1 (AIC=14428.74, BIC=14608.81). Given that Model 1 was nested within Model 2 (i.e., the less complex model was nested within the more complex model), the Satorra-Bentler scaled chi-square difference test was used to compare the two models [9]. Using loglikelihood values and scaling correction factors obtained from the maximum likelihood robust estimator, the chi-square test indicated a difference between the two models. Model 2 was superior in fit (TRd=28.98, *p*<.01); however, extremely high correlations were observed among the three factors in Model 2 (*r*s=.90-.99, *p*s<.01). These high intercorrelations may cause collinearity problems and suggest that the three factors are not distinct; rather, a single factor may be the best representation of the data. Given these considerations, Model 1 (1 first-order factor, unidimensional) was selected.

**Reliability**

Following identification of the unidimensional factor structure, item loadings were examined. Standardized loadings for all 15 items exceeded the recommended loading of 0.40 (see S9 Appendix); thus, all items were considered to contribute to the overall measurement of self-compassion. The internal consistency reliability of the scale was found to be excellent (α=0.95).

**Construct validity**

Construct validity of the SCI was demonstrated through significant correlations with other variables theoretically associated with self-compassion (see S10 Appendix). Specifically, the SCI was positively associated with mindfulness (i.e., acting with awareness, nonjudging, and nonreactivity), quality of life, peaceful acceptance of cancer, active coping, and progress in values-based living. Additionally, the SCI was negatively associated with depressive symptoms, anxiety, rumination, denial, struggle with illness, psychological inflexibility, cognitive fusion, and obstruction in values-based living. A moderate positive correlation was also found between the SCI and the existing SCS-SF.

Further evidence for the construct validity of the SCI was obtained. Compared to associations with the SCS-SF, associations were significantly smaller between the SCI and anxiety, rumination, denial, struggle with illness, psychological inflexibility, cognitive fusion, and obstruction in values-based living (see S10 Appendix). Additionally, compared to associations with negative items of the SCS-SF, associations were significantly smaller between the SCI and depressive symptoms and anxiety.

**References**

1. Tabachnick BG, Fidell LS. Using multivariate statistics. 6th ed. Needham Heights, MA: Allyn & Bacon; 2012.

2. Hayton JC, Allen DG, Scarpello V. Factor retention decisions in exploratory factor analysis: a tutorial on parallel analysis. Organ Res Methods. 2004;7(2):191-205.

3. Muthén LK, Muthén BO. Mplus user’s guide. 8th ed. Los Angeles, CA: Muthén & Muthén; 1998-2017.

4. Jia F, Wu W. Evaluating methods for handling missing ordinal data in structural equation modeling. Behav Res Methods. 2019;51(5):2337-55.

5. West SG, Taylor AB, Wu W. Model fit and model selection in structural equation modeling. In: Hoyle RH, editor. Handbook of structural equation modeling. New York: Guilford Press; 2012. pp. 209-231.

6. Presaghi F, Desimoni M. random.polychor.pa: A parallel analysis with polychoric correlation matrices. R package version 1.1.4-4; 2020.

7. Neff K. Self-compassion: An alternative conceptualization of a healthy attitude toward oneself. Self Identity. 2003;2(2):85-101.

8. Steiger JH. Tests for comparing elements of a correlation matrix. Psychol Bull. 1980;87(2):245.

9. Satorra A, Bentler PM. Ensuring positiveness of the scaled difference chi-square test statistic. Psychometrika. 2010;75(2):243-8.
