# Supplementary material for: Development and preliminary validation of the Brief Self-Compassion Inventory
Source: PLoS One. 2023 May 12;18(5):e0285658. doi: 10.1371/journal.pone.0285658 (PMC10180635; doi:10.1371/journal.pone.0285658)
Supplement: S5 Appendix — (DOCX) [file pone.0285658.s005.docx]

**S5 Appendix. Descriptive Statistics for Self-Compassion Inventory Items (*N*=372).**

| **Item** | **Mean** | **SD** | **Item-Total Correlation** |
| --- | --- | --- | --- |
| 1. I was kind to myself even when I was going through a tough time. | 3.68 | 1.14 | 0.66 |
| 2. Knowing that others have faced challenges similar to mine gave me courage. | 3.78 | 1.16 | 0.65 |
| 3. I noticed my difficult feelings without dwelling on them. | 3.45 | 1.13 | 0.60 |
| 4. When I noticed my flaws, I remembered that nobody is perfect. | 3.71 | 1.17 | 0.72 |
| 5. I was patient and understanding towards myself when I faced challenges. | 3.67 | 0.99 | 0.72 |
| 6. I accepted my thoughts and feelings without needing to change them. | 3.57 | 1.03 | 0.73 |
| 7. Even though I’ve failed before, I gave myself some slack. | 3.58 | 1.05 | 0.74 |
| 8. I recognized that others experience times of stress like I do. | 4.00 | 1.04 | 0.70 |
| 9. When I had difficult feelings, I realized that these feelings would change over time. | 3.76 | 1.09 | 0.78 |
| 10. I experienced my painful thoughts and feelings instead of trying to avoid them. | 3.55 | 1.11 | 0.66 |
| 11. When I faced a challenge, I reminded myself that challenges are a part of every human life. | 3.97 | 1.06 | 0.75 |
| 12. I forgave myself for my mistakes. | 3.76 | 1.07 | 0.72 |
| 13. I recognized that my struggles are also experienced by others. | 4.02 | 1.05 | 0.77 |
| 14. I was able to soothe myself during times of stress. | 3.67 | 1.00 | 0.73 |
| 15. I accepted my painful thoughts and feelings as a natural part of life. | 3.85 | 1.05 | 0.73 |
