# Supplementary material for: Development and preliminary validation of the Brief Self-Compassion Inventory
Source: PLoS One. 2023 May 12;18(5):e0285658. doi: 10.1371/journal.pone.0285658 (PMC10180635; doi:10.1371/journal.pone.0285658)
Supplement: S6 Appendix — (DOCX) [file pone.0285658.s006.docx]

**S6 Appendix.**

**Inter-item Pearson Correlations for the Self-Compassion Inventory (*N*=404).**

|  | **1** | **2** | **3** | **4** | **5** | **6** | **7** | **8** | **9** | **10** | **11** | **12** | **13** | **14** | **15** |
| --- | --- | --- | --- | --- | --- | --- | --- | --- | --- | --- | --- | --- | --- | --- | --- |
| 1 | -- |  |  |  |  |  |  |  |  |  |  |  |  |  |  |
| 2 | 0.52 | -- |  |  |  |  |  |  |  |  |  |  |  |  |  |
| 3 | 0.51 | 0.43 | -- |  |  |  |  |  |  |  |  |  |  |  |  |
| 4 | 0.50 | 0.49 | 0.55 | -- |  |  |  |  |  |  |  |  |  |  |  |
| 5 | 0.59 | 0.46 | 0.48 | 0.57 | -- |  |  |  |  |  |  |  |  |  |  |
| 6 | 0.56 | 0.43 | 0.47 | 0.54 | 0.73 | -- |  |  |  |  |  |  |  |  |  |
| 7 | 0.51 | 0.49 | 0.44 | 0.62 | 0.62 | 0.67 | -- |  |  |  |  |  |  |  |  |
| 8 | 0.42 | 0.54 | 0.46 | 0.58 | 0.47 | 0.51 | 0.50 | -- |  |  |  |  |  |  |  |
| 9 | 0.53 | 0.57 | 0.50 | 0.59 | 0.54 | 0.57 | 0.63 | 0.65 | -- |  |  |  |  |  |  |
| 10 | 0.48 | 0.49 | 0.49 | 0.49 | 0.43 | 0.50 | 0.51 | 0.53 | 0.64 | -- |  |  |  |  |  |
| 11 | 0.46 | 0.50 | 0.42 | 0.58 | 0.53 | 0.55 | 0.52 | 0.58 | 0.63 | 0.55 | -- |  |  |  |  |
| 12 | 0.50 | 0.44 | 0.36 | 0.54 | 0.57 | 0.54 | 0.62 | 0.48 | 0.56 | 0.43 | 0.64 | -- |  |  |  |
| 13 | 0.47 | 0.59 | 0.44 | 0.60 | 0.49 | 0.49 | 0.52 | 0.71 | 0.65 | 0.49 | 0.70 | 0.67 | -- |  |  |
| 14 | 0.47 | 0.45 | 0.43 | 0.50 | 0.58 | 0.57 | 0.57 | 0.48 | 0.56 | 0.48 | 0.64 | 0.62 | 0.61 | -- |  |
| 15 | 0.52 | 0.48 | 0.43 | 0.50 | 0.55 | 0.54 | 0.54 | 0.47 | 0.57 | 0.48 | 0.63 | 0.64 | 0.65 | 0.74 | -- |

All correlations are significant at *p*<.01.

**Inter-item Polychoric Correlations for the Self-Compassion Inventory (*N*=404).**

|  | **1** | **2** | **3** | **4** | **5** | **6** | **7** | **8** | **9** | **10** | **11** | **12** | **13** | **14** | **15** |
| --- | --- | --- | --- | --- | --- | --- | --- | --- | --- | --- | --- | --- | --- | --- | --- |
| 1 | -- |  |  |  |  |  |  |  |  |  |  |  |  |  |  |
| 2 | 0.60 | -- |  |  |  |  |  |  |  |  |  |  |  |  |  |
| 3 | 0.57 | 0.50 | -- |  |  |  |  |  |  |  |  |  |  |  |  |
| 4 | 0.57 | 0.56 | 0.62 | -- |  |  |  |  |  |  |  |  |  |  |  |
| 5 | 0.66 | 0.53 | 0.55 | 0.65 | -- |  |  |  |  |  |  |  |  |  |  |
| 6 | 0.63 | 0.50 | 0.54 | 0.61 | 0.80 | -- |  |  |  |  |  |  |  |  |  |
| 7 | 0.58 | 0.56 | 0.52 | 0.68 | 0.69 | 0.74 | -- |  |  |  |  |  |  |  |  |
| 8 | 0.49 | 0.61 | 0.54 | 0.66 | 0.54 | 0.59 | 0.59 | -- |  |  |  |  |  |  |  |
| 9 | 0.61 | 0.63 | 0.57 | 0.67 | 0.61 | 0.64 | 0.69 | 0.72 | -- |  |  |  |  |  |  |
| 10 | 0.54 | 0.56 | 0.55 | 0.57 | 0.50 | 0.57 | 0.58 | 0.61 | 0.70 | -- |  |  |  |  |  |
| 11 | 0.56 | 0.58 | 0.50 | 0.67 | 0.61 | 0.63 | 0.60 | 0.66 | 0.72 | 0.64 | -- |  |  |  |  |
| 12 | 0.57 | 0.50 | 0.43 | 0.60 | 0.63 | 0.61 | 0.68 | 0.55 | 0.63 | 0.49 | 0.72 | -- |  |  |  |
| 13 | 0.55 | 0.66 | 0.51 | 0.68 | 0.56 | 0.56 | 0.60 | 0.78 | 0.72 | 0.57 | 0.78 | 0.73 | -- |  |  |
| 14 | 0.54 | 0.51 | 0.50 | 0.58 | 0.65 | 0.64 | 0.64 | 0.54 | 0.64 | 0.54 | 0.72 | 0.68 | 0.68 | -- |  |
| 15 | 0.59 | 0.55 | 0.50 | 0.57 | 0.62 | 0.61 | 0.62 | 0.56 | 0.65 | 0.55 | 0.72 | 0.70 | 0.73 | 0.81 | -- |
