# Supplementary material for: Development and preliminary validation of the Brief Self-Compassion Inventory
Source: PLoS One. 2023 May 12;18(5):e0285658. doi: 10.1371/journal.pone.0285658 (PMC10180635; doi:10.1371/journal.pone.0285658)
Supplement: S7 Appendix — (DOCX) [file pone.0285658.s007.docx]

**S7 Appendix. Exploratory Factor Analysis (EFA) and Parallel Analysis Results for the 15-Item Self-Compassion Inventory.**

**Table 1.s7**

EFA Results

|  | **One-Factor Model** | **Two-Factor Model** | | **Three-Factor Model** | | |
| --- | --- | --- | --- | --- | --- | --- |
|  | **Factor** | **Factor 1** | **Factor 2** | **Factor 1** | **Factor 2** | **Factor 3** |
| **Item 1** | **0.67*** | **0.56*** | 0.17 | **0.76*** | -0.02 | -0.12 |
| **Item 2** | **0.66*** | 0.13 | **0.57*** | **0.50*** | 0.08 | 0.21 |
| **Item 3** | **0.62*** | **0.40*** | 0.26 | **0.80*** | -0.18 | 0.01 |
| **Item 4** | **0.75*** | **0.34*** | **0.45*** | **0.73*** | -0.01 | 0.09 |
| **Item 5** | **0.74*** | **0.85*** | -0.02 | **0.87*** | 0.06 | **-0.32*** |
| **Item 6** | **0.76*** | **0.84*** | 0.01 | **0.95*** | -0.03 | **-0.28*** |
| **Item 7** | **0.77*** | **0.63*** | 0.20 | **0.82*** | 0.03 | -0.14 |
| **Item 8** | **0.74*** | -0.01 | **0.78*** | **0.56*** | 0.01 | 0.41* |
| **Item 9** | **0.81*** | 0.20 | **0.65*** | **0.62*** | 0.09 | 0.23 |
| **Item 10** | **0.68*** | 0.24 | **0.47*** | 0.64 | -0.03 | 0.16 |
| **Item 11** | **0.79*** | 0.06 | **0.77*** | 0.11 | **0.61*** | **0.23*** |
| **Item 12** | **0.77*** | 0.28 | **0.53*** | 0.13 | **0.68*** | 0.01 |
| **Item 13** | **0.79*** | -0.21 | **1.04*** | 0.003 | **0.63*** | **0.44*** |
| **Item 14** | **0.77*** | 0.35 | **0.46*** | -0.001 | **0.87*** | -0.08 |
| **Item 15** | **0.77*** | 0.26 | **0.55*** | -0.07 | **0.90*** | -0.003 |
| **Correlations between the factors** |  | r_12_ = .77* | | r_12_ = .87*, r_13_ = .44, r_23_ = .35 | | |
| **Model fit** | *χ*^2^(90) = 339.19, RMSEA = 0.08[.07, .09], CFI = .89, SRMR = .05 | *χ*^2^(76) = 234.11, RMSEA = 0.07[.06, .08], CFI = .93, SRMR = .04 | | *χ*^2^(63) = 162.51, RMSEA = 0.06[.05, .07], CFI = .96, SRMR = .03 | | |

* Significant at *p* < .05. CFI = comparative fit index. RMSEA = root-mean-square error of approximation. SRMR = standardized root mean square residual.

**Figure 1.s7**

Parallel Analysis Plot with Pearson Correlations.


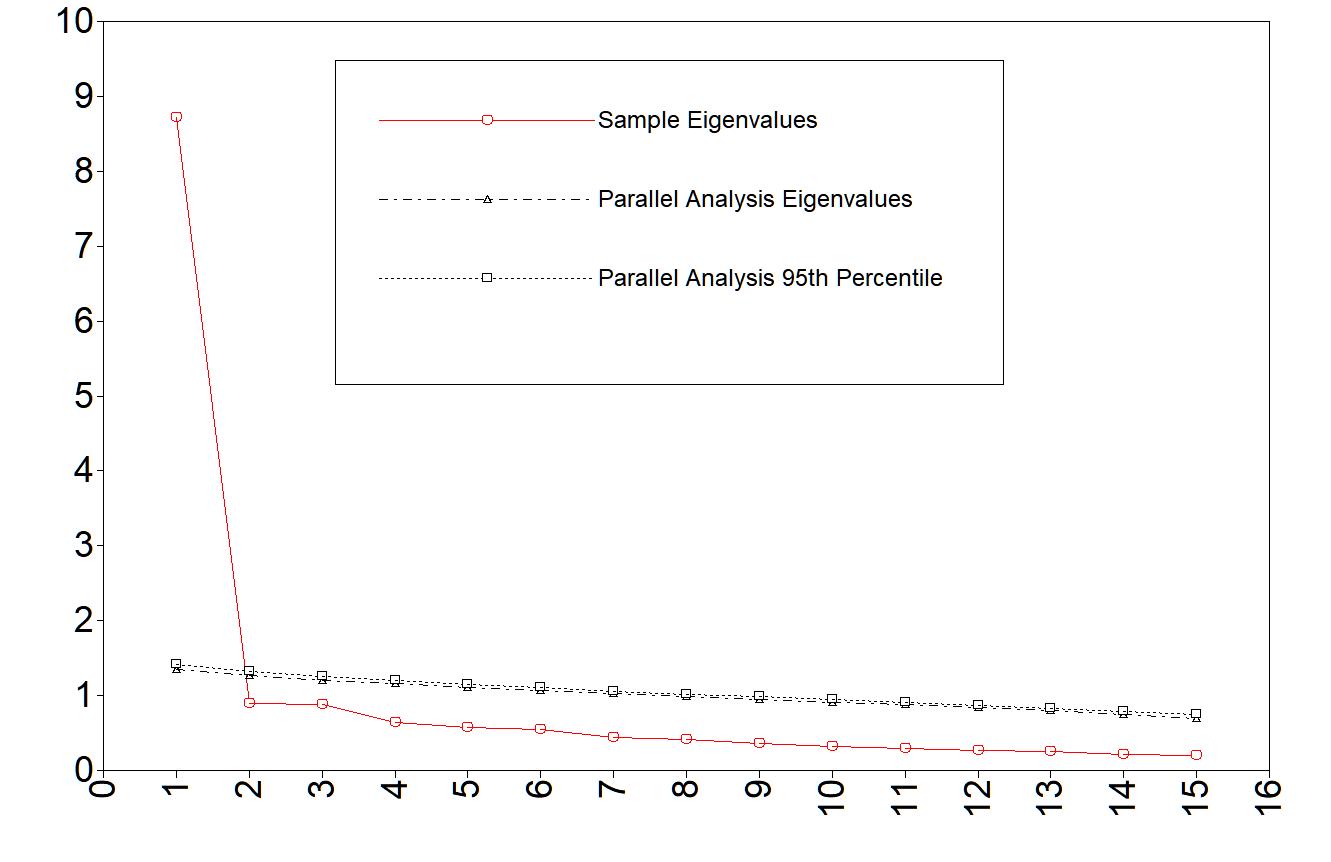


**Parallel Analysis Syntax and Results Based on Polychoric Correlations**

The parallel analysis was conducted using the random.polychor.pa package in R.

poly.mat(sc)

random.polychor.pa(nrep=1000, data.matrix=sc.data,comparison = "bootstrap",q.eigen=.95)

Comparison between BOOTSTRAP eigenvalues and EMPIRICAL eigenvalues

******* RESULTS for PARALLEL ANALYSIS:

# of factors (PCA) for PA method (Polychoric Corr.)...........: 1

# of factors for PA method (Polychoric Corr.).................: 1
