# Supplementary material for: Development and preliminary validation of the Brief Self-Compassion Inventory
Source: PLoS One. 2023 May 12;18(5):e0285658. doi: 10.1371/journal.pone.0285658 (PMC10180635; doi:10.1371/journal.pone.0285658)
Supplement: S8 Appendix — (DOCX) [file pone.0285658.s008.docx]

**S8 Appendix. Goodness of Fit Indices for Proposed Factor Structures for the 15-Item Self-Compassion Inventory.**

| **Model** | **Model Description** | **Fit Indices** | | | | |
| --- | --- | --- | --- | --- | --- | --- |
|  |  | **SRMR** | **CFI** | **RMSEA** | **AIC** | **BIC** |
| 1 | 1 first-order factor (unidimensional) | 0.05 | 0.89 | 0.08 | 14428.74 | 14608.81 |
| 2 | 1 second-order factor, 3 first-order factors | 0.05 | 0.91 | 0.08 | 14370.94 | 14563.01 |

SRMR = standardized root mean square residual. CFI = comparative fit index. RMSEA = root mean square error of approximation. AIC = Akaike information criterion. BIC = Bayesian information criterion.
