# Supplementary material for: Development and preliminary validation of the Brief Self-Compassion Inventory
Source: PLoS One. 2023 May 12;18(5):e0285658. doi: 10.1371/journal.pone.0285658 (PMC10180635; doi:10.1371/journal.pone.0285658)
Supplement: S9 Appendix — (DOCX) [file pone.0285658.s009.docx]

**S9 Appendix. Item Loadings for the Self-Compassion Inventory.**

| **Item** | **Standardized Loadings** |
| --- | --- |
| 1. I was kind to myself even when I was going through a tough time. | 0.67 |
| 2. Knowing that others have faced challenges similar to mine gave me courage. | 0.66 |
| 3. I noticed my difficult feelings without dwelling on them. | 0.62 |
| 4. When I noticed my flaws, I remembered that nobody is perfect. | 0.75 |
| 5. I was patient and understanding towards myself when I faced challenges. | 0.74 |
| 6. I accepted my thoughts and feelings without needing to change them. | 0.76 |
| 7. Even though I’ve failed before, I gave myself some slack. | 0.77 |
| 8. I recognized that others experience times of stress like I do. | 0.74 |
| 9. When I had difficult feelings, I realized that these feelings would change over time. | 0.81 |
| 10. I experienced my painful thoughts and feelings instead of trying to avoid them. | 0.68 |
| 11. When I faced a challenge, I reminded myself that challenges are a part of every human life. | 0.79 |
| 12. I forgave myself for my mistakes. | 0.77 |
| 13. I recognized that my struggles are also experienced by others. | 0.79 |
| 14. I was able to soothe myself during times of stress. | 0.77 |
| 15. I accepted my painful thoughts and feelings as a natural part of life. | 0.77 |
