# Supplementary material for: Development and preliminary validation of the Brief Self-Compassion Inventory
Source: PLoS One. 2023 May 12;18(5):e0285658. doi: 10.1371/journal.pone.0285658 (PMC10180635; doi:10.1371/journal.pone.0285658)
Supplement: S10 Appendix — (DOCX) [file pone.0285658.s010.docx]

**S10 Appendix. Correlations for Assessing the Construct Validity of the 15-Item Self-Compassion Inventory.**

|  | ***n*** | **SCI** | **SCS-SF Total** | **SCS-SF Positive Items** | **SCS-SF Negative Items** | ***z***^a^ | ***p*** | ***z***^b^ | ***p*** |
| --- | --- | --- | --- | --- | --- | --- | --- | --- | --- |
| Acting with awareness | 396 | 0.21* | 0.44* | 0.20* | 0.51* | -- | -- | -- | -- |
| Nonjudging | 391 | 0.16* | 0.45* | 0.18* | 0.54* | -- | -- | -- | -- |
| Nonreactivity | 397 | 0.35* | 0.16* | 0.34* | -0.05 | -- | -- | -- | -- |
| Quality of life | 403 | 0.40* | 0.47* | 0.28* | 0.48* | -- | -- | -- | -- |
| Peaceful acceptance of illness | 397 | 0.38* | 0.46* | 0.34* | 0.40* | -- | -- | -- | -- |
| Active coping | 401 | 0.18* | 0.09 | 0.26* | -0.08 | -- | -- | -- | -- |
| Values-based living - Progress | 398 | 0.50* | 0.57* | 0.56* | 0.40* | -- | -- | -- | -- |
| Depressive symptoms | 390 | -0.39* | -0.50* | -0.30* | -0.52* | 1.91 | 0.06 | 2.29 | 0.02 |
| Anxiety symptoms | 397 | -0.36* | -0.49* | -0.27* | -0.52* | 2.23 | 0.03 | 2.80 | 0.01 |
| Rumination | 390 | -0.33* | -0.68* | -0.38* | -0.72* | 6.76 | 0.00 | -- | -- |
| Denial | 397 | -0.17* | -0.30* | -0.13* | -0.35* | 2.09 | 0.04 | -- | -- |
| Struggle with illness | 398 | -0.30* | -0.52* | -0.26* | -0.58* | 3.59 | 0.00 | -- | -- |
| Psychological inflexibility | 396 | -0.40* | -0.61* | -0.30* | -0.67* | 4.00 | 0.00 | -- | -- |
| Cognitive fusion | 397 | -0.38* | -0.70* | -0.38* | -0.75* | 6.56 | 0.00 | -- | -- |
| Values-based living - Obstruction | 402 | -0.37* | -0.61* | -0.32* | -0.65* | 4.53 | 0.00 | -- | -- |
| SCS-SF Total | 390 | 0.58* | -- | -- | -- | -- | -- | -- | -- |

SCI = Self-Compassion Inventory. SCS-SF = Self-Compassion Scale–Short Form. No estimated correlation coefficients are reported between the SCS-SF Total score, SCS-SF Positive Items, and SCS-SF Negative Items, as all three cannot be simultaneously entered in the model due to collinearity issues.

**p*<.05.

^a^Statistically comparing correlations between negative variables and the BSCI vs. the SCS-SF.

^b^Statistically comparing correlations between depressive and anxiety symptoms and the BSCI vs. negative items of the SCS-SF.
