# Supplementary material for: Development and preliminary validation of the Brief Self-Compassion Inventory
Source: PLoS One. 2023 May 12;18(5):e0285658. doi: 10.1371/journal.pone.0285658 (PMC10180635; doi:10.1371/journal.pone.0285658)
Supplement: S11 Appendix — (DOCX) [file pone.0285658.s011.docx]

**S11 Appendix. Code for Data Analyses.**

**Mplus Code for Exploratory Factor Analysis with 1-3 factors for the 15-Item Self-Compassion Inventory**

DATA:

FILE IS scidata.csv;

VARIABLE:

NAMES ARE

new_sc1 new_sc2 new_sc3 new_sc4 new_sc5

new_sc6 new_sc7 new_sc8 new_sc9

new_sc10 new_sc11 new_sc12

new_sc13 new_sc14 new_sc15

;

USEVARIABLES ARE

new_sc1 new_sc2 new_sc3 new_sc4 new_sc5

new_sc6 new_sc7 new_sc8 new_sc9

new_sc10 new_sc11 new_sc12

new_sc13 new_sc14 new_sc15;

missing are all (999);

ANALYSIS:

TYPE = EFA 1 3;

ESTIMATOR = MLr;

ROTATION = geomin(ob);

PLOT:

TYPE = PLOT2;

**Mplus Code for Parallel Analysis for the 15-Item Self-Compassion Inventory**

TITLE:

Exploratory Factor Analysis (EFA)

DATA:

FILE IS scidata.csv;

VARIABLE:

NAMES ARE

new_sc1 new_sc2 new_sc3 new_sc4 new_sc5

new_sc6 new_sc7 new_sc8 new_sc9

new_sc10 new_sc11 new_sc12

new_sc13 new_sc14 new_sc15

;

USEVARIABLES ARE

new_sc1 new_sc2 new_sc3 new_sc4 new_sc5

new_sc6 new_sc7 new_sc8 new_sc9

new_sc10 new_sc11 new_sc12

new_sc13 new_sc14 new_sc15;

missing are all (999);

!!!!!!!!!!!!!!!!!!!!!!!!!!!!!!!!!!!!!!!!!!!!!!!!!!!!!!!!!!!!!!!!!!!!!!!!!!!!!!!!!!

! "PARALLEL =" indicates parallel analysis for EFA.

! "1000" means 1000 random data sets that is drawn.

!

! CAUTION:

! 1. Only maximum likelihood estimation is available for parallel analysis.

! 2. When you conduct parallel analysis, you cannot specify ANY factor rotation method.

!!!!!!!!!!!!!!!!!!!!!!!!!!!!!!!!!!!!!!!!!!!!!!!!!!!!!!!!!!!!!!!!!!!!!!!!!!!!!!!!!!

ANALYSIS:

TYPE = EFA 1 2;

ESTIMATOR = MLr;

PARALLEL = 1000;

PLOT:

TYPE = PLOT2;

! draw the scree plot with eigenvalues from parallel analysis.

**R Code for Reliability Coefficients and Confirmatory Factor Analysis of the 15-item Self-Compassion Inventory and 5-item Brief Self-Compassion Inventory**

library(foreign)

library(psych)

library(ggplot2)

library(lavaan)

library(semPlot)

library(semTools)

library(GPArotation)

library(psychTools)

library(random.polychor.pa)

# READ IN DATA

setwd("C:/specify location of data here")

data1 <-read.spss(file="ACT survey data 12.6.19.sav",

to.data.frame = TRUE,

use.value.labels = FALSE)

names(data1)

list <- c("new_sc1", "new_sc2", "new_sc3",

"new_sc4", "new_sc5", "new_sc6",

"new_sc7", "new_sc8", "new_sc9",

"new_sc10", "new_sc11", "new_sc12",

"new_sc13", "new_sc14", "new_sc15")

sc <- data1[list]

describe(sc)

# CFA and Calculate reliability coefficients (alpha and omega) for the full version

cfa1 <- '

sci =~ NA*new_sc1 + new_sc2 + new_sc3 +

new_sc4 + new_sc5 + new_sc6 +

new_sc7 + new_sc8 + new_sc9 +

new_sc10 + new_sc11 + new_sc12 +

new_sc13 + new_sc14 + new_sc15

sci ~~ 1*sci

'

fit.1 <- cfa(model = cfa1, data = sc , missing = 'ML', estimator = 'MLR')

summary(fit.1, fit.measures = TRUE, standardized = TRUE, rsquare = TRUE)

semPaths(fit.1, intercepts = FALSE)

reliability(fit.1)

# CFA and Calculate reliability coefficients (alpha and omega) for the short version

cfa2 <- '

sci =~ NA*new_sc9 + new_sc11 + new_sc13 + new_sc14 + new_sc15

sci ~~ 1*sci

'

fit.2 <- cfa(model = cfa2, data = sc , missing = 'ML', estimator = 'MLR')

summary(fit.2, fit.measures = TRUE, standardized = TRUE, rsquare = TRUE)

semPaths(fit.2, intercepts = FALSE)

reliability(fit.2)

**R Code for Parallel Analysis of a Pearson Correlation Matrix and Polychoric Correlation Matrix for the 15-Item Self-Compassion Inventory**

# output Pearson correlation matrix

names(sc) <- c( "sc1" , "sc2", "sc3" , "sc4" , "sc5" , "sc6" , "sc7" ,

"nsc8", "sc9" , "sc10", "sc11", "sc12", "sc13", "sc14", "sc15")

round(cor(sc, method = "pearson", use = "complete.obs"), 2)

# output polychoric correlation matrix

sc<-na.exclude(as.matrix(sc))

polychoric(sc)

# Parallel analysis of Pearson Correlations

fa.parallel(sc, fa = "fa", fm = "ml")

fa.parallel(sc, fa = "pc", fm = "ml")

# parallel analysis of Polychoric Correlations

random.polychor.pa(nrep=100, data.matrix=sc,comparison = "bootstrap",q.eigen=.95)

**MPlus Code for Correlations Between the 5-item Brief Self-Compassion Inventory and Psychological Variables**

Title:conval_kc_sf5 (5 items only)

Data:File IS "convaldata.dat";

Variable:

NAMES ARE

study_id age gender CancerType CancerStage new_sc1 new_sc2 new_sc3 new_sc4 new_sc5 new_sc6 new_sc7 new_sc8 new_sc9 new_sc10 new_sc11 new_sc12 new_sc13 new_sc14 new_sc15 Accept COPE FFMQNonReact FFMQNonJudge FFMQActAware FFMQTotal Ruminate VQObs VQProg Fusion PStruggle PAccept Pain Sleep Anxiety Depression CAMindful Fatigue FSI Social PainTScore SleepTScore AnxietyTScore DepressTScore AvoidCope ActiveCope Stage4 scstotal activecoping denial qol1 newsctotal;

USEVARIABLES ARE

scstotal Accept FFMQNonReact FFMQNonJudge FFMQActAware Ruminate VQObs VQProg Fusion PStruggle PAccept Anxiety Depression activecoping denial qol1 newscsf;

MISSING ARE all (-99);

Define:

newscsf = new_sc9 + new_sc11 + new_sc13 + new_sc14 + new_sc15;

analysis:

estimator = mlr;

model:

scstotal - newscsf with

scstotal - newscsf;

;

scstotal - newscsf;

output:

standardized;

**MPlus Code for Correlations between Positive and Negative Self-Compassion Scale-Short Form Items, the 5-item Brief Self-Compassion Inventory, and Psychological Variables**

Title: Correlations between pos- / neg-only SCS items and 5-item newsc-sf

Data:File IS "conval_scsraw.dat";

Variable:

NAMES ARE

study_id age gender CancerType CancerStage qol1 new_sc1 new_sc2 new_sc3 new_sc4 new_sc5 new_sc6 new_sc7 new_sc8 new_sc9 new_sc10 new_sc11 new_sc12 new_sc13 new_sc14 new_sc15 scs1 scs2 scs3 scs4 scs5 scs6 scs7 scs8 scs9 scs10 scs11 scs12 Accept COPE FFMQNonReact FFMQNonJudge FFMQActAware FFMQTotal Ruminate VQObs VQProg Fusion PStruggle PAccept Pain Sleep Anxiety Depression CAMindful Fatigue FSI Social ActiveCope Stage4 scs1R scs4R scs8R scs9R scs11R scs12R scstotal activecoping denial newsctotal;

USEVARIABLES ARE

Accept FFMQNonReact FFMQNonJudge FFMQActAware Ruminate VQObs VQProg Fusion PStruggle PAccept Anxiety Depression activecoping denial qol1 newscsf scspos scsneg;

MISSING ARE all (-99);

Define:

! summing 5 items to form the newsc short-form

newscsf = new_sc9 + new_sc11 + new_sc13 + new_sc14 + new_sc15;

! summing positively-worded SCS items ONLY (compassionate responding)

scspos = scs2 + scs3 +scs5 + scs6 + scs7 +scs10;

! summing negatively-worded SCS items ONLY (uncompassionate responding)

scsneg = scs1R + scs4R + scs8R + scs9R + scs11R + scs12R;

analysis:

estimator = mlr;

model:

Accept - scsneg with

Accept - scsneg;

Accept - scsneg;

output:

standardized;

**MPlus Code for Correlations between the Self-Compassion Scale-Short Form Total Scores and Psychological Variables**

Title: Correlations between SCS total scores and psych variables

Data:File IS "conval_scsraw.dat";

Variable:

NAMES ARE

study_id age gender CancerType CancerStage qol1 new_sc1 new_sc2 new_sc3 new_sc4 new_sc5 new_sc6 new_sc7 new_sc8 new_sc9 new_sc10 new_sc11 new_sc12 new_sc13 new_sc14 new_sc15 scs1 scs2 scs3 scs4 scs5 scs6 scs7 scs8 scs9 scs10 scs11 scs12 Accept COPE FFMQNonReact FFMQNonJudge FFMQActAware FFMQTotal Ruminate VQObs VQProg Fusion PStruggle PAccept Pain Sleep Anxiety Depression CAMindful Fatigue FSI Social ActiveCope Stage4 scs1R scs4R scs8R scs9R scs11R scs12R scstotal activecoping denial newsctotal;

USEVARIABLES ARE

Accept FFMQNonReact FFMQNonJudge FFMQActAware Ruminate VQObs VQProg Fusion PStruggle PAccept Anxiety Depression activecoping denial qol1 scstotal;

MISSING ARE all (-99);

analysis:

estimator = mlr;

model:

Accept - scstotal with

Accept - scstotal;

Accept - scstotal;

output:

standardized;

**MPlus Code for Correlations between Positive and Negative Self-Compassion Scale-Short Form Items, the 15-item Self-Compassion Inventory, and Psychological Variables**

Title: Correlations between pos- / neg-only SCS items and 15-item newsc

Data:File IS "conval_scsraw.dat";

Variable:

NAMES ARE

study_id age gender CancerType CancerStage qol1 new_sc1 new_sc2 new_sc3 new_sc4 new_sc5 new_sc6 new_sc7 new_sc8 new_sc9 new_sc10 new_sc11 new_sc12 new_sc13 new_sc14 new_sc15 scs1 scs2 scs3 scs4 scs5 scs6 scs7 scs8 scs9 scs10 scs11 scs12 Accept COPE FFMQNonReact FFMQNonJudge FFMQActAware FFMQTotal Ruminate VQObs VQProg Fusion PStruggle PAccept Pain Sleep Anxiety Depression CAMindful Fatigue FSI Social ActiveCope Stage4 scs1R scs4R scs8R scs9R scs11R scs12R scstotal activecoping denial newsctotal;

USEVARIABLES ARE

Accept FFMQNonReact FFMQNonJudge FFMQActAware Ruminate VQObs VQProg Fusion PStruggle PAccept Anxiety Depression activecoping denial qol1 newsctotal scspos scsneg;

MISSING ARE all (-99);

Define:

! summing positively-worded SCS items ONLY (compassionate responding)

scspos = scs2 + scs3 +scs5 + scs6 + scs7 +scs10;

! summing negatively-worded SCS items ONLY (uncompassionate responding)

scsneg = scs1R + scs4R + scs8R + scs9R + scs11R + scs12R;

analysis:

estimator = mlr;

model:

Accept - scsneg with

Accept - scsneg;

Accept - scsneg;

output:

standardized;

**MPlus Code for Measurement Invariance Testing of the 5-item Brief Self-Compassion**

**Inventory Across Cancer Stage (Configural, Metric, and Scalar)**

DATA: FILE IS "invar.dat";

VARIABLE:

NAMES ARE

study_id age gender CancerType CancerStage Stage4 new_sc1 new_sc2 new_sc3 new_sc4 new_sc5 new_sc6 new_sc7 new_sc8 new_sc9 new_sc10 new_sc11 new_sc12 new_sc13 new_sc14 new_sc15;

USEVARIABLES

new_sc9 new_sc11 new_sc13 new_sc14 new_sc15;

GROUPING IS Stage4 (0=early 1=advanced);

MISSING ARE all (-99);

Analysis:

Estimator = MLR;

Model = configural metric scalar;

MODEL:

SC by

new_sc9*

new_sc11

new_sc13

new_sc14

new_sc15;

SC@1;

OUTPUT:

tech1;

standardized;

**MPlus Code for Measurement Invariance Testing of the 5-item Brief Self-Compassion Inventory Across Cancer Stage (Strict Invariance)**

DATA: FILE IS "invar.dat";

VARIABLE:

NAMES ARE

study_id age gender CancerType CancerStage Stage4 new_sc1 new_sc2 new_sc3 new_sc4 new_sc5 new_sc6 new_sc7 new_sc8 new_sc9 new_sc10 new_sc11 new_sc12 new_sc13 new_sc14 new_sc15;

USEVARIABLES

new_sc9 new_sc11 new_sc13 new_sc14 new_sc15;

GROUPING IS Stage4 (0=ely 1=adv);

MISSING ARE all (-99);

Analysis:

Estimator = MLR;

MODEL:

! Early stage reference group model

SC by

new_sc9* new_sc11 new_sc13 new_sc14 new_sc15;

SC@1; ! Factor variance fixed to equal 1

[SC@0]; ! Factor mean fixed to equal 0

new_sc9 new_sc11 new_sc13 new_sc14 new_sc15 (1-5);

MODEL adv:

! Advanced stage group alternative model

new_sc9 new_sc11 new_sc13 new_sc14 new_sc15 (1-5);

OUTPUT:

tech1;

stdyx;

**MPlus Code for Measurement Invariance Testing of the 5-item Brief Self-Compassion Inventory Across Cancer Type (Configural, Metric, and Scalar Invariance)**

TITLE: 1f_cantype_all_sf5 (5 items)

DATA: FILE IS "invar.dat";

VARIABLE:

NAMES ARE

study_id age gender CancerType CancerStage Stage4 new_sc1 new_sc2 new_sc3 new_sc4 new_sc5 new_sc6 new_sc7 new_sc8 new_sc9 new_sc10 new_sc11 new_sc12 new_sc13 new_sc14 new_sc15;

USEVARIABLES

new_sc9 new_sc11 new_sc13 new_sc14 new_sc15;

GROUPING IS CanType (1=breast 2=prostate 3=lung 4=gi);

MISSING ARE all (-99);

Analysis:

Estimator = MLR;

Model = configural metric scalar;

MODEL:

SC by

new_sc9*

new_sc11

new_sc13

new_sc14

new_sc15;

SC@1;

OUTPUT:

tech1;

standardized;

**MPlus Code for Measurement Invariance Testing of the 5-item Brief Self-Compassion Inventory Across Cancer Type (Strict Invariance)**

DATA: FILE IS "invar.dat";

VARIABLE:

NAMES ARE

study_id age gender CancerType CancerStage Stage4 new_sc1 new_sc2 new_sc3 new_sc4 new_sc5 new_sc6 new_sc7 new_sc8 new_sc9 new_sc10 new_sc11 new_sc12 new_sc13 new_sc14 new_sc15;

USEVARIABLES

new_sc9 new_sc11 new_sc13 new_sc14 new_sc15;

GROUPING IS CanType (1=breast 2=prostate 3=lung 4=gi);

MISSING ARE all (-99);

Analysis:

Estimator = MLR;

MODEL:

! Breast reference group model

SC by

new_sc9* new_sc11 new_sc13 new_sc14 new_sc15;

SC@1; ! Factor variance fixed to equal 1

[SC@0]; ! Factor mean fixed to equal 0

new_sc9 new_sc11 new_sc13 new_sc14 new_sc15 (1-5);

MODEL prostate:

! Prostate group alternative model

new_sc9 new_sc11 new_sc13 new_sc14 new_sc15 (1-5);

MODEL lung:

! Lung group alternative model

new_sc9 new_sc11 new_sc13 new_sc14 new_sc15 (1-5);

MODEL gi:

! Gi group alternative model

new_sc9 new_sc11 new_sc13 new_sc14 new_sc15 (1-5);

OUTPUT:

tech1 stdyx;

**MPlus Code for Measurement Invariance Testing of the 5-item Brief Self-Compassion Inventory Across Gender (Configural, Metric, and Scalar Invariance)**

DATA: FILE IS "invar.dat";

VARIABLE:

NAMES ARE

study_id age gender CancerType CancerStage Stage4 new_sc1 new_sc2 new_sc3 new_sc4 new_sc5 new_sc6 new_sc7 new_sc8 new_sc9 new_sc10 new_sc11 new_sc12 new_sc13 new_sc14 new_sc15;

USEVARIABLES

new_sc9 new_sc11 new_sc13 new_sc14 new_sc15;

GROUPING IS gender (1=male 2=female);

MISSING ARE all (-99);

Analysis:

Estimator = MLR;

Model = configural metric scalar;

MODEL:

SC by

new_sc9*

new_sc11

new_sc13

new_sc14

new_sc15;

SC@1;

OUTPUT:

tech1;

standardized;

**MPlus Code for Measurement Invariance Testing of the 5-item Brief Self-Compassion Inventory Across Gender (Strict Invariance)**

DATA: FILE IS "invar.dat";

VARIABLE:

NAMES ARE

study_id age gender CancerType CancerStage Stage4 new_sc1 new_sc2 new_sc3 new_sc4 new_sc5 new_sc6 new_sc7 new_sc8 new_sc9 new_sc10 new_sc11 new_sc12 new_sc13 new_sc14 new_sc15;

USEVARIABLES

new_sc9 new_sc11 new_sc13 new_sc14 new_sc15;

GROUPING IS gender (1=male 2=female);

MISSING ARE all (-99);

Analysis:

Estimator = MLR;

MODEL:

! Male reference group model

SC by

new_sc9* new_sc11 new_sc13 new_sc14 new_sc15;

SC@1; ! Factor variance fixed to equal 1

[SC@0]; ! Factor mean fixed to equal 0

new_sc9 new_sc11 new_sc13 new_sc14 new_sc15 (1-5);

MODEL female:

! Female group alternative model

new_sc9 new_sc11 new_sc13 new_sc14 new_sc15 (1-5);

OUTPUT:

tech1;
